# Comparative Studies on Phenolic Composition, Antioxidant, Wound Healing and Cytotoxic Activities of Selected *Achillea* L. Species Growing in Turkey

**DOI:** 10.3390/molecules201017976

**Published:** 2015-09-30

**Authors:** Osman Tuncay Agar, Miris Dikmen, Nilgun Ozturk, Mustafa Abdullah Yilmaz, Hamdi Temel, Fatma Pinar Turkmenoglu

**Affiliations:** 1 Department of Pharmaceutical Botany, Faculty of Pharmacy, Hacettepe University, Ankara 06100, Turkey; E-Mail: tagar@hacettepe.edu.tr; 2 Department of Pharmacology, Faculty of Pharmacy, Anadolu University, Eskisehir 26470, Turkey; E-Mail: mirisd@anadolu.edu.tr; 3 Department of Pharmacognosy, Faculty of Pharmacy, Anadolu University, Eskisehir 26470, Turkey; E-Mail: nozturk@anadolu.edu.tr; 4 Dicle University Science and Technology Research and Application Center, Diyarbakır 21280, Turkey; E-Mails: mustafaabdullahyilmaz@gmail.com (M.A.Y.); htemel@dicle.edu.tr (H.T.); 5 Department of Pharmaceutical Chemistry, Faculty of Pharmacy, Dicle University, Diyarbakir 21280, Turkey

**Keywords:** *Achillea*, phenolics, LC-MS/MS, antioxidant, wound healing, cytotoxic, breast cancer, natural product

## Abstract

Turkey is one of the most important centers of diversity for the genus *Achillea* L. in the world. Keeping in mind the immense medicinal importance of phenols, in this study, three species growing in Turkey, *A. coarctata* Poir. (AC), *A. kotschyi* Boiss. subsp. *kotschyi* (AK) and *A. lycaonica* Boiss. & Heldr. (AL) were evaluated for their phenolic compositions, total phenolic contents (TPC), antioxidant properties, wound healing potencies on NIH-3T3 fibroblasts and cytotoxic effects on MCF-7 human breast cancer cells. Comprehensive LC-MS/MS analysis revealed that AK was distinctively rich in chlorogenic acid, hyperoside, apigenin, hesperidin, rutin, kaempferol and luteolin (2890.6, 987.3, 797.0, 422.5, 188.1, 159.4 and 121.2 µg analyte/g extract, respectively). The findings exhibited a strong correlation between TPC and both free radical scavenging activity and total antioxidant capacity (TAC). Among studied species, the highest TPC (148.00 mg GAE/g extract) and TAC (2.080 UAE), the strongest radical scavenging (EC_50_ = 32.63 μg/mL), the most prominent wound healing and most abundant cytotoxic activities were observed with AK. The results suggested that AK is a valuable source of flavonoids and chlorogenic acid with important antioxidant, wound healing and cytotoxic activities. These findings warrant further studies to assess the potential of AK as a bioactive source that could be exploited in pharmaceutical, cosmetics and food industries.

## 1. Introduction

The genus *Achillea* L. (Asteraceae), which is commonly known as “yarrow”, is represented by more than 140 perennial herbaceous species mainly distributed in the Northern hemisphere. The name of the genus originates from the name of “Achilles” from Greek mythology since he used yarrow to treat his bleeding ankle and wounds. Besides their use as wound healing agents, *Achillea* species have been used in folk medicine worldwide for numerous other medicinal applications for thousands of years [1]. Today, some therapeutic applications, such as antinociceptive, antiinflammatory [2,3], wound healing [4,5,6], antispasmodic and hepatoprotective [7,8] uses, are approved for several *Achillea* species by scientific experimental results. Some *Achillea* species are also consumed as vegetables, spices, beverages and additives in the food and cosmetic industry and in horticulture [1].

Previous phytochemical studies have revealed that the genus *Achillea* is rich in terpenoids and phenolics, with phenolic acids, flavonoids and lignans being possible bioactive compounds [1,9,10]. Flavonoids, which are the most diverse and prevalent compounds ubiquitous in all plant organs, constitute the most important group of natural phenolics [11]. In the genus *Achillea*, flavonoids mainly occur as flavones and flavonols and their derivatives. *Achillea* flavonoids are usually mono- and diglycosides of apigenin, luteolin and quercetin. Besides flavonoid glycosides, many of the yarrow species also accumulate free flavonoid aglycons. Methyl and methoxy derivatives were also found in these species [9,10,12,13,14,15]. In addition to flavonoids, hydroxycinnamic acids such as chlorogenic, caffeic, dicaffeoylquinic, and hydroxybenzoic acids such as vanillic and syringic acids were also qualified and quantified in some yarrow species [13,14,15,16,17].

Phenolic compounds are one of the most important groups acting as free radical terminators and primary antioxidants in medicinal plants and functional foods [11,18,19]. Free radicals are continuously produced in the human body as normal products of cellular metabolism. Over-production of free radicals, exposure to external oxidant substances or a failure in the regulation mechanisms, may cause damage to valuable biomolecules such as DNA, lipids and proteins in our body. This damage contributes to development of many diseases including cancer, Alzheimer’s disease, Parkinson’s disease, aging, diabetes and atherosclerosis [20,21]. It is also well known that the normal physiology of wound healing depends on low levels of reactive oxygen species and oxidative stress, and an overexposure to oxidative stress leads to impaired wound healing [22,23]. The role of natural antioxidants, especially plant phenolics, in preventing or delaying degenerative diseases caused by free radicals is well documented [18,19,24,25]. Moreover, natural antioxidants are generally more desirable for consumption than synthetic ones which have shown potential health risks and toxicity, most notably possible carcinogenic efects. Therefore, in order to find new sources of safe antioxidants of natural origin which could be used in pharmaceutical preparations, cosmetics and foods, it is vital to learn about the amounts and varieties of phenolic compounds in medicinal plants and plant extracts [24,25].

Turkey is one of the main centers of diversity for the genus *Achillea*. A total of 54 *Achillea* taxa are indigenous to Turkey, of which 31 are endemic (endemism ratio, 57%) [1]. Although infusions and decoctions prepared from aerial parts of many species of the genus are widely used topically for wounds and internally for various purposes in Turkish folk medicine [1,26,27,28], scientific data is limited. To overcome the lack of information, in this comparative study, we aimed to investigate phenolic composition and biological activities of methanol extracts from three *Achillea* species growing in Turkey, *A. coarctata* Poir. (AC), *A. kotschyi* Boiss. subsp. kotschyi (AK) and *A. lycaonica* Boiss. & Heldr. (AL). For this aim, an effective and robust ESI-MS/MS method was applied for the determination and quantification of phenolic compounds. Total phenolic contents of the extracts were measured, and *in vitro* antioxidant activities were examined by using different assays. Furthermore, the wound healing potential of these species on NIH-3T3 fibroblast cells was evaluated. According to Cragg and Newman’s report, as some visible skin conditions such as wart, callus and abscesses may correspond to cancerous conditions, plants used for skin disorders might have anticancerous potential [29]. In the light of this literature, finally, we investigated the cytotoxic potential of the extracts against MCF-7 cells as breast cancer is listed among the most common cause of cancer death in the world [30]. To the best of our knowledge, this study provides the first data on the phenolic profile, antioxidant capacity, wound healing potential and cytotoxic activity against MCF-7 cells of methanol extracts of AC, AK and AL in the literature.

## 2. Results and Discussion

### 2.1. Identification and Quantification of Phenolic Compounds

**Figure 1:**
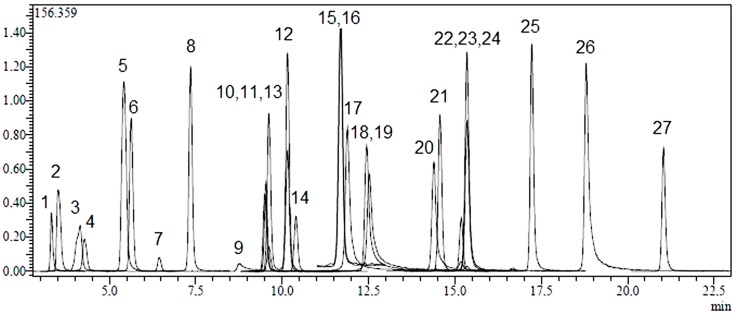
LC-MS/MS chromatograms of 250 ppb standard mix. Notes: Chromatographic conditions were given in Experimental section. Standard compounds: 1: quinic acid, 2: malic acid, 3: tr-aconitic acid, 4: gallic acid, 5: chlorogenic acid, 6: protocatechuic acid, 7: tannic acid, 8: tr-caffeicacid, 9: vanillin, 10: *p*-coumaric acid, 11: rosmarinic acid, 12: rutin, 13: hesperidin, 14: hyperoside, 15: 4-OH benzoic acid, 16: salicylic acid, 17: myricetin, 18: fisetin, 19: coumarin, 20: quercetin, 21: naringenin, 22: hesperetin, 23: luteolin, 24: kaempferol, 25: apigenin, 26: rhamnetin, 27: chrysin.

**Figure 2:**
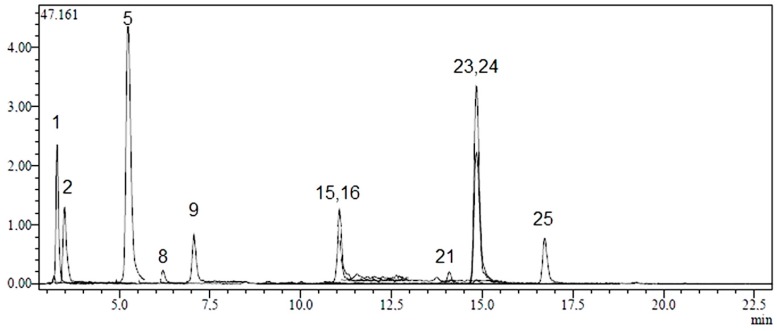
LC-MS/MS chromatograms of AC. Notes: Chromatographic conditions were given in Experimental Section.

**Figure 3:**
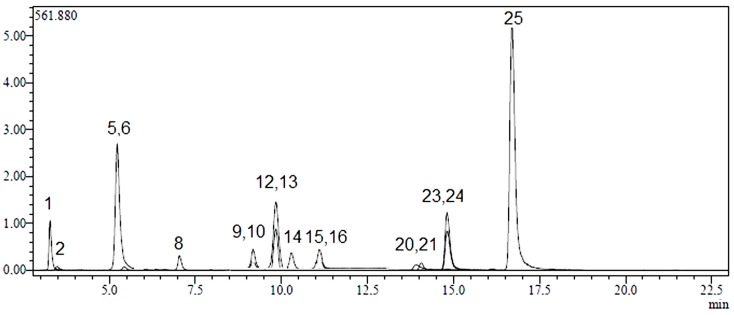
LC-MS/MS chromatograms of AK. Notes: Chromatographic conditions were given in the Experimental Section.

**Figure 4:**
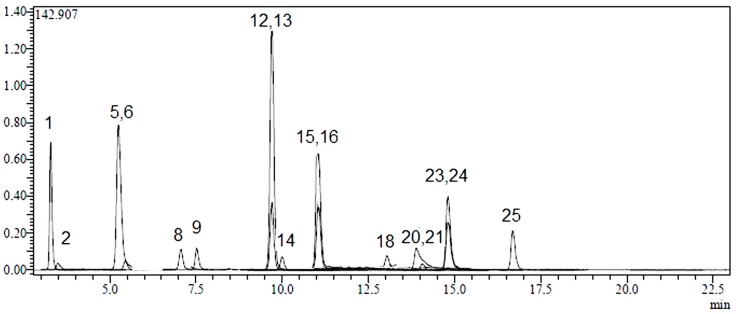
LC-MS/MS chromatograms of AL. Notes: Chromatographic conditions were given in the Experimental Section.

Twenty-four phenolic compounds (flavonoids, flavonoid glycosides, hydroxycinnamic and hydroxybenzoic acids) as well as three non-phenolic organic acids which are widespread in plant materials were analysed by an LC-MS/MS method. The applicability of the proposed analytical method and the qualitative and quantitative determination of the standard compounds have already been verified [31,32]. LC-MS/MS chromatograms of standard compounds, AC, AK and AL extracts are presented in Figure 1, Figure 2, Figure 3 and Figure 4, respectively. The concentrations of identified compounds analyzed in *Achillea* extracts are given in Table 1. They were shown in the order of their retention time.

It was determined that in AC extract, chlorogenic acid was the most abundant phenolic compound (511.9 ± 2.51 µg analyte/g extract). Small concentrations of vanillin, salicylic, tr-caffeic and 4-OH-benzoic acids were also quantified (40.5 ± 2.0 µg analyte/g extract, 10.1 ± 0.5 µg analyte/g extract, 9.5 ± 0.5 µg analyte/g extract and 4.2 ± 0.2 µg analyte/g extract, respectively). In terms of flavonoids, AC contained kaempferol, luteolin, apigenin and naringenin in low amounts (45.0 ± 2.3 µg analyte/g extract, 29.8 ± 2.1 µg analyte/g extract, 10.8 ± 0.6 µg analyte/g extract and 2.6 ± 0.1 µg analyte/g extract, respectively) (Figure 2 and Table 1).

**Table 1:** Analytical parameters of LC-MS/MS method, and identification and quantification of phenolic compounds in AC, AK and AL.

No	Analyte	Parent Ion (*m*/*z*) ^a^	MS^2^ (CE) ^b^	RT ^c^	R^2^ ^d^	RSD% ^e^	Linearity Range (mg/L)	LOD/LOQ (µg/L) ^f^	Recovery (%)	Quantification (µg Analyte/g Extract) ^g^		
										AC	AK	AL
1	Quinic acid	190.95	85 (22), 93 (22)	3.32	0.9927	0.0388	250–10,000	22.3/74.5	103.3	596.6 ± 28.6	2545.6 ± 122	16214.8 ± 778.3
2	Malic acid	133.05	115 (14), 71 (17)	3.54	0.9975	0.1214	250–10,000	19.2/64.1	101.4	127.6 ± 6.8	80.5 ± 4.3	40.7 ± 2.2
3	tr-Aconitic acid	172.85	85 (12), 129 (9)	4.13	0.9933	0.3908	250–10,000	15.6/51.9	102.8	N.D. ^h^	N.D.	N.D.
4	Gallic acid	169.05	125 (14), 79 (25)	4.29	0.9901	0.4734	25–1000	4.8/15.9	102.3	N.D.	N.D.	N.D.
5	Chlorogenic acid	353	191 (17)	5.43	0.9932	0.1882	250–10,000	7.3/24.3	99.7	511.9 ± 25.1	2890.6 ± 141.6	778.0 ± 38.1
6	Protocatechuic acid	152.95	109 (16), 108 (26)	5.63	0.9991	0.5958	100–4000	25.8/85.9	100.2	N.D.	53.7 ± 2.7	32.8±1.7
7	Tannic acid	182.95	124 (22), 78 (34)	6.46	0.9955	0.9075	100–4000	10.2/34.2	97.8	N.D.	N.D.	N.D.
8	tr-Caffeic acid	178.95	135 (15), 134 (24), 89 (31)	7.37	0.9942	1.0080	25–1000	4.4/14.7	98.6	9.5 ± 0.5	34.9 ± 1.8	12.9 ± 0.7
9	Vanillin	151.05	136 (17), 92 (21)	8.77	0.9995	0.4094	250–10,000	10.1/33.7	99.2	40.5 ± 2.0	66.0 ± 3.2	75.5 ± 3.7
10	*p*-Coumaric acid	162.95	119 (15), 93 (31)	9.53	0.9909	1.1358	100–4000	15.2/50.8	98.4	N.D.	13.6 ± 0.7	N.D.
11	Rosmarinic acid	358.9	161 (17), 133 (42)	9.57	0.9992	0.5220	250–10,000	10.4/34.8	101.7	N.D.	N.D.	N.D.
12	Rutin	609.1	300 (37), 271 (51), 301 (38)	10.18	0.9971	0.8146	250–10,000	17.0/56.6	102.2	N.D.	188.1 ± 9.4	442.2 ± 22.1
13	Hesperidin	611.1	303 (24), 465 (12)	9.69	0.9973	0.1363	250–10,000	21.6/71.9	100.2	N.D.	422.5 ± 20.7	1267.3 ± 62.1
14	Hyperoside	463.1	300 (27), 301 (26)	10.43	0.9949	0.2135	100–4000	12.4/41.4	98.5	N.D.	987.3 ± 48.4	167.3 ± 8.2
15	4-OH Benzoic acid	136.95	93 (17), 65 (27)	11.72	0.9925	1.4013	25–1000	3.0/10.0	106.2	4.2 ± 0.2	21.2 ± 1.1	22.0 ± 1.1
16	Salicylic acid	136.95	93 (16), 65 (31), 75 (30)	11.72	0.9904	0.6619	25–1000	4/13.3	106.2	10.1 ± 0.5	32.9 ± 1.6	34.2 ± 1.7
17	Myricetin	317	179 (19), 151(23), 137 (26)	11.94	0.9991	2.8247	100–4000	9.9/32.9	106.0	N.D.	N.D.	N.D.
18	Fisetin	284.95	135 (22), 121 (27)	12.61	0.9988	2.4262	100–4000	10.7/35.6	96.9	N.D.	N.D.	23.2 ± 1.3
19	Coumarin	146.95	103 (17), 91 (26), 77 (27)	12.52	0.9924	0.4203	100–4000	9.1/30.4	104.4	N.D.	N.D.	N.D.
20	Quercetin	300.9	179 (19), 151 (21), 121 (28)	14.48	0.9995	4.3149	25–1000	2.0/6.8	98.9	N.D.	28.5 ± 2.0	27.8 ± 2.0
21	Naringenin	270.95	151 (18), 119 (24), 107 (26)	14.66	0.9956	2.0200	25–1000	2.6/8.8	97.0	2.6 ± 0.1	22.3 ± 1.2	4.3 ± 0.2
22	Hesperetin	300.95	164 (25), 136 (33), 108 (42)	15.29	0.9961	1.0164	25–1000	3.3/11.0	102.4	N.D.	N.D.	N.D.
23	Luteolin	284.95	217 (25), 199 (28), 175 (29)	15.43	0.9992	3.9487	25–1000	5.8/19.4	105.4	29.8 ± 2.1	121.2 ± 8.4	31.5 ± 2.2
24	Kaempferol	284.95	217 (29), 133 (32), 151 (23)	15.43	0.9917	0.5885	25–1000	2.0/6.6	99.1	45.0 ± 2.3	159.4 ± 8.3	50.0 ± 2.6
25	Apigenin	268.95	151 (25), 117 (35)	17.31	0.9954	0.6782	25–1000	0.1/0.3	98.9	10.8 ± 0.6	797.0 ± 39.9	28.5 ± 1.51
26	Rhamnetin	314.95	165 (23), 121 (28), 300 (22)	18.94	0.9994	2.5678	25–1000	0.2/0.7	100.8	N.D.	N.D.	N.D.
27	Chrysin	253	143 (29), 119 (32), 107 (26)	21.18	0.9965	1.5530	25–1000	0.05/0.17	102.2	N.D.	N.D.	N.D.

^a^ Parent ion (*m*/*z):* Molecular ions of the standard compounds (mass to charge ratio); ^b^ MS^2^ (CE): MRM fragments for the related molecular ions (CE refers to related collision energies of the fragment ions); ^c^ RT: Retention time; ^d^ R^2^: coefficient of determination; ^e^ RSD: relative standard deviation; ^f^ LOD/LOQ (µg/L): Limit of detection/Limit of quantification; ^g^ Values in µg/g (*w*/*w*) of plant extract; ^h^ N.D.: not detected.

AK extract had a very rich phenolic content because of its high levels of flavonoids such as hyperoside (987.3 ± 48.4 µg analyte/g extract), apigenin (797.0 ± 39.9 µg analyte/g extract), hesperidin (422.5 ± 20.7 µg analyte/g extract), rutin (188.1 ± 9.4 µg analyte/g extract), kaempferol (159.4 ± 8.3 µg analyte/g extract) and luteolin (121.2 ± 8.4 µg analyte/g extract), and significant amount of chlorogenic acid (2890.6 ± 141.6 µg analyte/g extract) as hydroxycinnamic acid derivative. (Figure 3 and Table 1).

AL extract had high levels of hesperidin (1267.3 ± 62.1 µg analyte/g extract), rutin (442.2 ± 22.1 µg analyte/g extract), hyperoside (167.3 ± 8.2 µg analyte/g extract) and chlorogenic acid (778.0 ± 38.1 µg analyte/g extract) (Figure 4 and Table 1).

In terms of non-phenolic compounds, AC, AK and AL contain high amounts of quinic acid (596.6 ± 28.6 µg analyte/g extract, 2545.6 ± 122 µg analyte/g extract and 16214.8 ± 778.3 µg analyte/g extract, respectively) and lower amounts of malic acid (127.6 ± 6.8 µg analyte/g extract, 80.5 ± 4.3 µg analyte/g extract, 40.7 ± 2.2 µg analyte/g extract, respectively) (Figure 2, Figure 3 and Figure 4 and Table 1). Among twenty-seven references used; tr-acotinic, gallic, tannic, rosmarinic acids, and myricetin, coumarin, hesperetin, rhamnetin and chrysin were not detected and/or quantified in all *Achillea* extracts employed in this study.

In the genus *Achillea*, as mentioned in the introduction, mono- and diglycosides of apigenin, luteolin and quercetin are the most abundant flavonoid glycosides. Many of the yarrow species also accumulate free flavonoid aglycons. Methyl and methoxy derivatives were also reported [9,10,12,13,14,15]. Besides flavonoids, hydoxycinnamic and hydrozybenzoic acids were also reported in some yarrow species [13,14,15,16,17]. In an HPLC-ESI-MS study on *A. ligustica*, it was shown that 6-hydroxykaempferol-3,6,4ʹ-trimethyl ether, apigenin-6-*C*-glucoside-8-*C*-arabinoside, luteolin, and apigenin were the most abundant flavonoids in the ethanolic extract of flowering tops [12]. Vitalini *et al.* [13] demonstrated that the phytochemical profile of *A. millefolium*, which is the officinal species of the genus *Achillea*, is mainly characterized by the presence of chlorogenic acid and its caffeoilquinic derivatives, besides rutin, luteolin and apigenin flavonoid glycosides. In addition to luteolin and its glucoside, presence of rutin, rosmarinic, chlorogenic and caffeic acids in *A. millefolium* were also reported [15]. In another study regarding two subspecies of *A. distans*, it was reported that the ethanolic extract of *A. distans* subsp. *distans* flowers contained high amounts of luteolin followed by apigenin and small amounts of quercetin. Caffeic and chlorogenic acids were also reported in low concentrations. In the extract of *A. distans* subsp. *alpina*, luteolin and apigenin were found in smaller amounts. Small amounts of quercetin and hyperoside was also reported in addition to very low concentrations of rutin and isoquercitrin [14]. If we look at our results in terms of mostly reported flavonoids, apigenin was quantified in high amount only in AK. Luteolin concentration was also higher in AK. Apigenin and luteolin were quantified in AC and AL at lower concentrations. Quercetin was not detected in AC, however, AK and AL contain it in small amounts. In addition to these findings, high amounts of hyperoside, hesperidin and rutin were detected in AK and AL, however, their quantities were different. Literature survey revealed that *Achillea* species contain hydroxycinnamic acids rather than hydroxybenzoic acids, and the most frequently reported hydroxycinnamic acids were chlorogenic and caffeic acids [13,14,15,16,17]. Chlorogenic acid was the most abundant hydroxycinnamic acid in AC, AK and AL, which is in good agreement with previously reported data. In terms of hydroxybenzoic acids, vanillic and syringic acids have previously been reported in *Achillea* [16]. In addition to previously reported hyroxybenzoic acids in *Achillea* species, we reported the presence of 4-OH-benzoic and salisilic acids in all species investigated and protocatechuic acid in AK and AL. It is well known that the genus *Achillea* shows a great chemical diversity [9,10], and this LC-MS/MS study well demonstrated qualitative and quantitative differences among phenolic compositions of AC, AK and AL. 

### 2.2. Total Phenolic Content

The yields of the methanol extracts as a percentage of plant material (*w*/*w*) were 8.88%, 5.85% and 6.19% for AC, AK and AL, respectively. Total phenolic content of extracts was determined by the Folin-Ciocalteu colorimetric method, based on the procedure of Singleton & Rossi [33] using gallic acid as a standard phenolic compound. It was suggested that Folin-Ciocalteu assay should be regarded as a measure of total antioxidant capacity rather than phenolic content as Folin-Ciocalteu reagent reacts with other antioxidants such as amino acids, proteins, vitamins and thiols. However, because phenolics are the most abundant antioxidants in most plants, Folin-Ciocalteu assay gives a good “ballpark” estimation of total phenolic content for most plants. Therefore, the assay has been used for many years by the food and agricultural industries and in plant sciences to determine the phenolic content of plant products [34].

Table 2 shows the total phenolic content and antioxidant activity of *Achillea* species employed in this study. The content of total phenols is expressed as gallic acid equivalents (mg GAE/g extract) and the values are given as means of triplicate analyses. Significantly, the highest amount of total phenols was determined to be 148.00 ± 0.92 mg GAE/g extract (*p* < 0.001) in AK, followed by AL (76.49 ± 1.67 mg GAE/g extract, *p* < 0.001). AC extract had the lowest total phenolic content (55.16 ± 0.96 mg GAE/g extract, *p* < 0.001).

**Table 2:** Total phenolic content and antioxidant activity of *Achillea* extracts *.

Sample	Total Phenolic Content	DPPH Scavenging Assay	β-Carotene-Linoleic Acid Assay	Total Antioxidant Capacity	
	mg GAE/g Extract ^1^	EC_50_ (μg/mL)	AA (%) ^2^	UAE ^3^ (mM)	CRE ^4^ (μM)
AC	55.16 ± 0.96 ^a^	94.09 ± 3.08^c^	74.12 ± 2.11 ^b^	1.038 ± 0.011^a^	2272.91 ± 25.18 ^a^
AK	148.00 ± 0.92 ^c^	32.63 ± 0.65 ^a^	60.06 ± 1.39^a^	2.080 ± 0.064 ^b^	4553.85 ± 140.37 ^b^
AL	76.49 ± 1.67 ^b^	70.01 ± 0.97 ^b^	81.46 ± 1.65 ^c^	1.250 ± 0.169 ^a^	2743.55 ± 369.59 ^a^
BHT	-	29.98 ± 1.15 ^a^	89.77 ± 0.99 ^d^	-	-

* Results are represented as means ± standard deviation (*n* = 3). Superscript letters in each column indicate significant difference according to Tukey honest significant test (*p* < 0.05); ^1^ Gallic acid equivalent; ^2^ Inhibition (%) = 100 × (1 − (As^0^ − As^180^)/(Ak^0^ − Ak^180^); ^3^ Uric acid equivalent; ^4^ Copper reducing equivalent.

In a previous study, total phenolic content of infusions prepared with the flower heads of AC and AK were reported to be 120 mg GAE/L infusion and 134 mg GAE/L infusion [35]. Phenolic contents in extracts obtained from other *Achillea* species were also reported. In an investigation on optimization of extraction parameters for assessing maximum yield of total phenolic content from *A. biebersteini* and *A. wilhelmsii* aerial parts, Bashi *et al.* reported minimum total phenolic contents in 80% methanol extracts prepared by simple maceration (20 mg GAE/g extract and 17 mg GAE/g extract, respectively) while maximum phenolic contents were determined in 80% methanol extracts prepared by ultrasound-assisted extraction at pH = 6.3 (117 mg GAE/g extract and 106 mg GAE/gextract, respectively) [36]. In another study, total phenolic contents were 118 mg GAE/g extract, 126 mg GAE/g extract and 134 mg GAE/g extract in 70% ethanol extracts of *A. aleppica* subsp. *aleppica*, *A. aleppica* subsp. *zederbaueri* and *A. biebesteinii* aerial parts, respectively [37]. Total phenolic content of infusions prepared with leaves and inflorescences of *A. collina* cultivars were reported to be 49 mg GAE/g extract and 31–32 mg GAE/g extract, respectively [38]. Although various parameters such as pretreatment of the sample prior to the extract, extraction method and other factors related plant species such as age, genetic factors, geographical location, season of harvesting, might be effective on the phenolic content determined by spectrophotometry, in light of these literature data, methanol extract of AK seems to contain significant amounts of phenolics.

### 2.3. Antioxidant Activity

#### 2.3.1. 1,1-Diphenyl-2-picrylhydrazyl (DPPH) Radical Scavenging Assay

Free radical scavenging activity of extracts was evaluated by DPPH method in comparison with that of a synthetic antioxidant, tert-butylhydoxy-toluene (BHT), at different concentrations [39]. Radical scavenger activity of the extracts was expressed as the amount of antioxidants necessary to decrease the initial DPPH absorbance by 50% (median effective concentration value, EC_50_) and results were given in Table 2. All extracts indicated significantly different scavenging properties (*p* < 0.001). Among the *Achillea* species, AK showed the highest DPPH radical scavenging ability (EC_50_ = 32.63 ± 0.65 μg/mL, *p* > 0.05 *vs.* BHT), which was similar to that of the reference compound BHT (EC_50_ = 29.98 ± 1.15 μg/mL). Based on EC_50_, the rank order was as follows: BHT > AK > AL > AC. The EC_50_ values of AL and AC were 70.01 ± 0.97 (*p* < 0.001 *vs.* BHT) and 94.09 ± 3.08 μg/mL (*p* < 0.001 *vs.* BHT), respectively.

#### 2.3.2. β-Carotene-Linoleic Acid Assay

The potential of *Achillea* extracts to inhibit lipid peroxidation was also evaluated in β-carotene-linoleic acid system [40]. There was also a highly significant statistical difference between the analyzed extracts in this system (*p* < 0.001). The decrease in absorbance of β-carotene in the extracts of *Achillea* and well-known antioxidant BHT, which was used as standard, is shown in Table 2. In this test system, it was obvious that AL oxidized most rapidly with % inhibition value of 81.46 ± 1.65 (*p* < 0.01 *vs.* BHT) after BHT (inhibition percentage: 89.77 ± 0.99). AK, which showed the most abundant DPPH radical scavenging activity, was the less effective one in β-carotene-linoleic acid system. The antioxidant capacity of the extracts by β-carotene-linoleic acid system decreased in the following order: BHT > AL > AC > AK.

#### 2.3.3. Total Antioxidant Capacity (TAC) Assay

The TAC assay, which is a single assay sufficient for reliable determination of antioxidant potential of a complex sample, is based on the reduction of copper (II) to copper (I) by antioxidants. Copper reducing antioxidant assays were reported to be simple and versatile antioxidant capacity assays useful for a wide variety of phenolics, including phenolic acids, flavonoids, carotenoids, anthocyanins, as well as for thiols, synthetic antioxidants, and Vitamins C and E [41]. TAC of *Achillea* extracts were shown as mM reducing equivalents to uric acid (UAE) and as μM copper reducing equivalents (CRE) in Table 2. According to our results, TAC of the extracts are in accordance with their total phenolic content. AK extract had the highest TAC (2.080 ± 0.064 UAE, *p* < 0.001), followed by AL (1.250 ± 0.169 UAE); AC extract had the lowest TAC (1.038 ± 0.011 UAE).

Antioxidant activity of infusions prepared with the flower heads of AC and AK (1 mg/mL) was previously evaluated by using different methods. Moderate protective effects of AC and AK infusions against H_2_O_2_-induced oxidative damage in human erythrocytes and leucocytes had been reported [42]. In another study performed by the same research group [35], DPPH radical scavenging capacity of the floral infusions of AC and AK were reported to be 23% and 27%, respectively, while OH**·** radical scavenging capacity of the same infusions were 35% and 37%, respectively. H_2_O_2_ reducing power of AC and AK infusions were found to be 40% and 44%, respectively. Total antioxidant capacity of infusions, based on the reduction of Mo (VI) to Mo (V), were determined as 4.671 and 5.599 mMα-Tocopherol/100mL. In the above mentioned study, similar to our results, infusions of AC were reported to have less DPPH scavenging ability and total antioxidant capacity [35]. However, as seen in Table 2, our results indicate significantly higher DPPH radical scavenging levels than reported for methanol extracts prepared with aerial parts of AC and AK. This might be due to higher phenolic content of the aerial parts than that of flowers. In a comparative study, infusions of leaves and inflorescences of two *A. collina* cultivars were investigated in terms of their phenolic content, free radical scavenging activity, *in vitro* cytotoxicity and lipid peroxidation in PC12 cells, and infusions of leaves had higher total phenolic contents, exhibited higher antioxidant properties and cytoprotective activity [38].

DPPH radical scavenging activity of *A. biebersteini* and *A. wilhelmsii* aerial parts were IC_50_ = 346 μg/mL and IC_50_ = 426 μg/mL, respectively, in 80% methanol macerations, and IC_50_ = 30 μg/mL and IC_50_ = 54 μg/mL, respectively, in ultrasound-assisted extracts [36]. Seventy percent of ethanol extracts of *A. aleppica* subsp. *aleppica*, *A. aleppica* subsp. *zederbaueri* and *A. biebesteinii* aerial parts showed DPPH radical scavenging activity with EC_50_ = 33 μg/mL, EC_50_ = 33 μg/mL and EC_50_ = 32 μg/mL, respectively [37]. When comparing our results with previous data, the strong DPPH radical scavenging activity of methanol extract of AK with a EC_50_ = 32 μg/mL was notable. As a general conclusion of previous investigations, it was indicated that high DPPH radical scavenging activity was observed in plant extracts which contain phenolic compounds in high amounts.

### 2.4. Correlation of Total Phenolic Content and Antioxidant Activity

As it is generally considered that, in *Achillea* extracts, phenolic compounds are the main bioactive compounds related to antioxidant activity [35,36,37,38], the correlation coefficient (*r*) was calculated in order to determine the relationship between phenolic content and antioxidant activity in three applied model systems. There was a statistically significant and strong positive correlation between phenolic content and both DPPH radical scavenging property (*r* = 0.982, *p* < 0.001) and total antioxidant capacity (*r* = 0.984, *p* < 0.001). Our results are in agreement with previous data in terms of correlation between total phenolic content of *Achillea* species and DPPH radical scavenging activity. Contrary to DPPH radical scavenging property, a negative strong correlation was calculated between total phenolic content and inhibitory effect in β-carotene bleaching system (*r* = −0.833, *p* < 0.01). The Folin-Ciocalteu method of performing a TPC has recently evolved as a total antioxidant capacity assay, but was found to be incapable of measuring lipophilic antioxidants due to the high affinity of the Folin-Ciocalteu chromophore toward water [43]. On the other hand, β-carotene bleaching assay only provides an indication of the level of lipophilic compounds because the β-carotene bleaching test is similar to an oil-in-water emulsion system [44]. All in all, besides phenolics, some other compounds having redox functional groups might contribute to the overall effect and influence the antioxidant properties exhibiting diverse solubility characteristics.

### 2.5. Effects on NIH-3T3 Mouse Fibroblast Cells

Fibroblasts, which are the major cellular component of the dermis regulating the skin physiology, are typically quiescent cells. In the proliferative phase of wound healing, fibroblasts produce various components of extracellular matrix including collagen, and then generate granulation tissue. Re-epithelialization occurred in this phase also involves in proliferation and migration of fibroblasts [45,46]. Quantification of fibroblast proliferation, migration and collagen synthesis in fibroblast cell cultures have first been proposed as a method for testing wound-healing activity *in vitro* by Graham *et al.* [47]. By using fibroblast cells of different origins, this approach has been successfully applied in studies on the development of new drugs and for the investigation of the mechanism of action [48,49,50,51].

**Figure 5:**
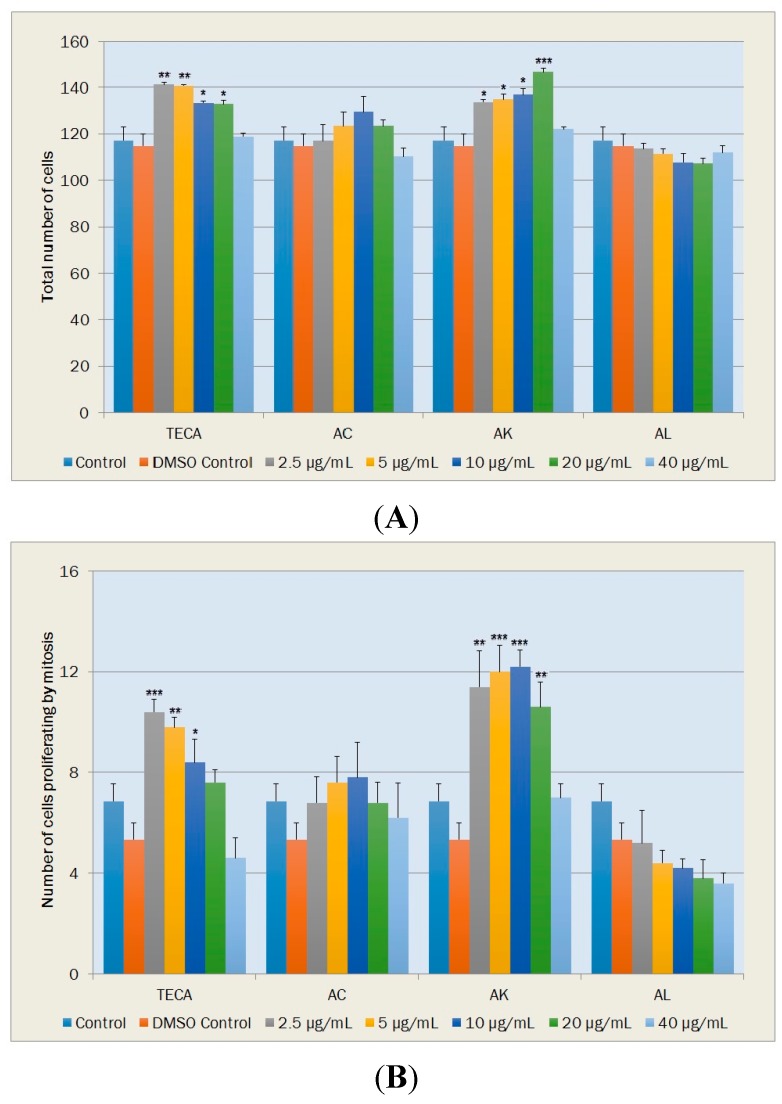
Effects of TECA, AC, AK and AL extracts on cell proliferation. (**A**) Total number of NIH-3T3 fibroblasts; (**B**) Total number of fibroblasts proliferating by mitosis Data are mean ± SEM values (cells/mm^2^, *n* = 5 in each case). * *p* < 0.05, ** *p* < 0.01, *** *p* < 0.001, significantly different from control values.

**Figure 6:**
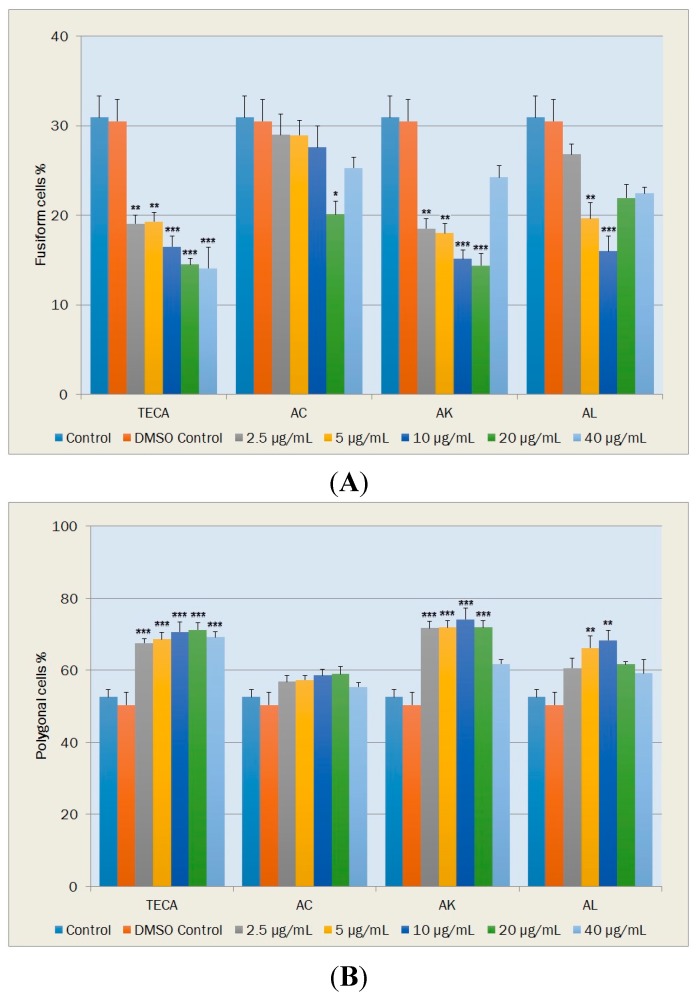
Effects of TECA, AC, AK and AL extracts on percentages of NIH-3T3 fibroblasts in fusiform (**A**); and polygonal (**B**) shapes. Data are mean ± SEM values (*n* = 5, in each case). * *p* < 0.05, ** *p* < 0.01, *** *p* < 0.001, significantly different from control values.

**Figure 7:**
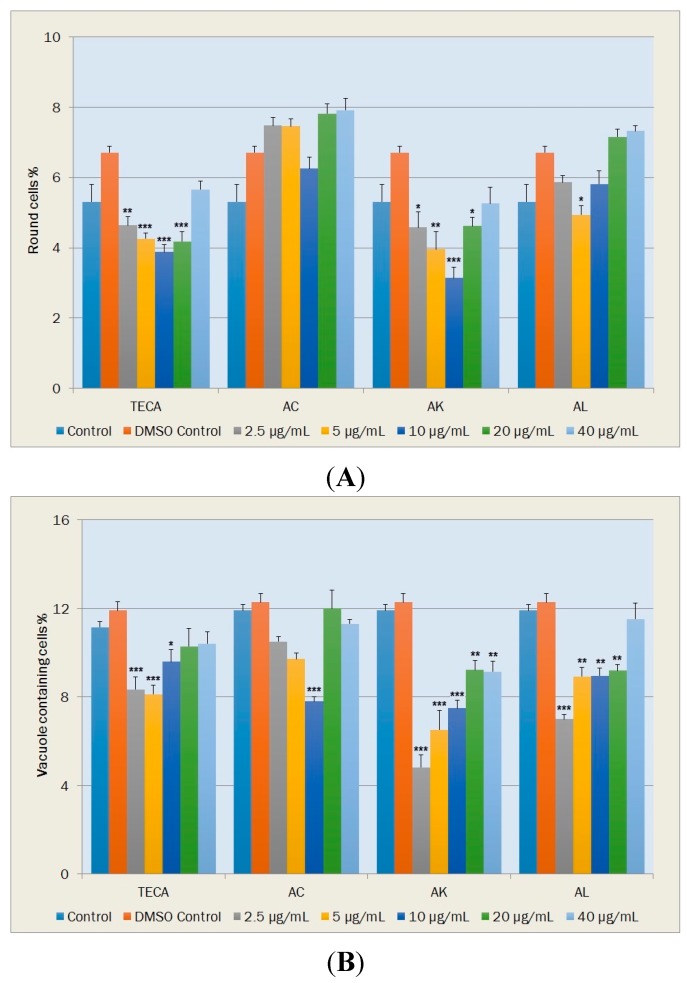
Effects of TECA, AC, AK and AL extracts on percentages of NIH-3T3 fibroblasts in round shape (**A**); and vacuole containing NIH-3T3 fibroblasts (**B**) Data are mean ± SEM values (*n* = 5, each case). * *p* < 0.05, ** *p* < 0.01, *** *p* < 0.001, significantly different from control values.

**Figure 8:**
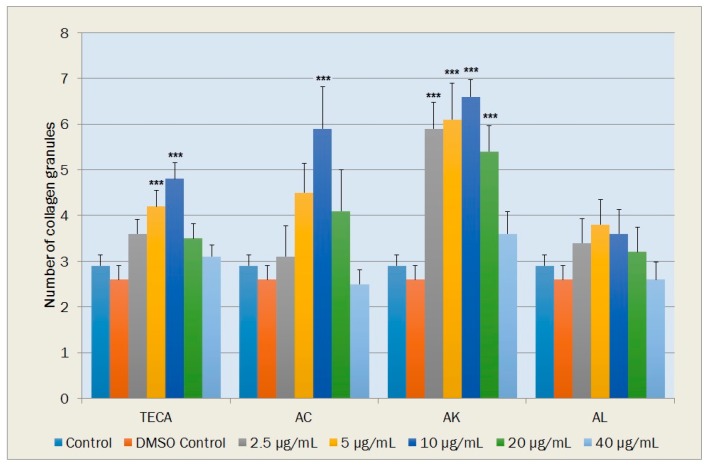
Effects of TECA, AC, AK and AL extracts on the number of collagen granules in NIH-3T3 fibroblasts. Data are mean ± SEM values (*n* = 10 in each case). *** *p* < 0.001, significantly different from control values.

In the present study, NIH-3T3 mouse fibroblast cells were treated with titrated extract of *Centella asiatica* (TECA) and AC, AK and AL extracts (at concentrations of 2.5, 5, 10, 20 and 40 μg/mL) for 24 h in growth medium. Controls and extract treated fibroblasts were stained and examined under a light microscope. In the light of previous studies [49,50,51], cell proliferation, morphological changes and collagen production in the fibroblasts were evaluated to approach possible wound repair mechanism(s). Results were evaluated in comparison to DMSO control, as it was used as a vehicle to dissolve TECA and *Achillea* extracts. Morphometric analyses performed in this study showed that there were no significant differences between the medium control groups and DMSO controls (*p* > 0.05). In addition, the DMSO concentration used here (0.2% *v*/*v*) was found to induce no significant negative effects on cultured NIH-3T3 cells (Figure 5, Figure 6, Figure 7 and Figure 8).

#### 2.5.1. Cell Proliferation

Total cell numbers and cell numbers under mitosis were used as parameters of proliferation of fibroblasts. Significant increases in total cell numbers were found with TECA at concentrations of 2.5, 5, 10 and 20 μg/mL when compared with DMSO control (*p* < 0.01, *p* < 0.01, *p* < 0.05 and *p* < 0.05, respectively) (Figure 5A). Among the *Achillea* extracts employed in this study, total cell numbers increased significantly only with AK at concentrations from 2.5 to 20 μg/mL (*p* < 0.05, *p* < 0.05, *p* < 0.05 and *p* < 0.001, respectively) (Figure 5A). Slight increases in the total cell numbers, which were observed with AC at concentrations from 2.5 to 20 μg/mL, were not statistically significant. AL did not increase the total number of cells in NIH-3T3 cultures (Figure 5A).

TECA also exhibited increases in mitotic cell numbers at concentrations from 2.5–20 μg/mL. Increases with TECA at concentrations of 2.5 μg/mL (*p* < 0.001), 5 μg/mL (*p* < 0.01) and 10 μg/mL (*p* < 0.05) were determined to be statistically significant relative to DMSO control (Figure 5B). Among tested samples, significant increases in mitotic cell numbers were observed only with AK at increasing concentrations from 2.5 to 20 μg/mL (*p* < 0.01, *p* < 0.001, *p* < 0.001 and *p* < 0.01, respectively) while a slight increase and a dose-dependent decrease in mitotic cell numbers were observed with AC and AL, respectively (Figure 5B).

#### 2.5.2. Morphological Changes and Vacuole Containing Cells

During tissue repair, fusiform fibroblasts undergo changes in phenotype from their normal quiescent state. Polygonal fibroblast cells are widely accepted as being the active form in wound healing since they have the ability to migrate and close wounds. Round fibroblasts, which are non-viabile cells, indicate cytotoxicity while vacuole containing cells are accepted as a measure for aging of the cell population. Percentages of these fibroblast cells were given in Figure 6 and Figure 7.

The percentages of fusiform cells significantly decreased at all TECA concentrations tested relative to DMSO control (*p* < 0.01~*p* < 0.001) (Figure 6A). Significant decreases in percentages of fusiform cells occurred with AC at the concentration of 20 μg/mL (*p* < 0.05); with AK at concentrations of 2.5, 5, 10 and 20 μg/mL (*p* < 0.01, *p* < 0.01, *p* < 0.001 and *p* < 0.001, respectively) and with AL at concentrations of 5 and 10 μg/mL (*p* < 0.01 and *p* < 0.001, respectively) (Figure 6A).

TECA, at tested concentrations, exhibited significant increases in the percentage of polygonal cells (*p* < 0.001) (Figure 6B). The percentage of polygonal cells was also increased with AK and AL at all tested concentrations. Statistically significant increases observed with AK at concentrations from 5–20 μg/mL (*p* < 0.001) and with AL at concentrations of 5 and 10 μg/mL (*p* < 0.01), relative to DMSO control (Figure 6B).

In terms of the percentage of round cells (Figure 7A), statistically significant decreases were determined with TECA at concentrations of 2.5, 5, 10 and 20 μg/mL (*p* < 0.01, *p* < 0.001, *p* < 0.001 and *p* < 0.001, respectively); with AK at concentrations of 2.5, 5, 10 and 20 μg/mL (*p* < 0.05, *p* < 0.01, *p* < 0.001 and *p* < 0.05, respectively) and with AL at the concentration of 5 μg/mL (*p* < 0.05) (Figure 7A).

The percentages of vacuole containing cells also exhibited significant decreases with TECA at concentrations of 2.5, 5 and 10 μg/mL (*p* < 0.001, *p* < 0.001 and *p* < 0.05, respectively) (Figure 7B). When comparing the DMSO control, significant decreases in the percentage of vacuole containing cells occurred with AC at the concentration of 10 μg/mL (*p* < 0.001); with AK at concentrations of 2.5, 5, 10, 20 and 40 μg/mL (*p* < 0.001, *p* < 0.001, *p* < 0.001, *p* < 0.01 and *p* < 0.01, respectively) and with AL at concentrations of 2.5, 5, 10 and 20 μg/mL (*p* < 0.001, *p* < 0.01, *p* < 0.01 and *p* < 0.01, respectively) (Figure 7B).

#### 2.5.3. Collagen Production in the Fibroblasts

The number of collagen granules in a cell was also determined as a sign of collagen production. In terms of the number of collagen granules, statistically significant increases relative to DMSO control were observed with TECA at concentrations of 5 and 10 μg/mL (*p* < 0.001); with AC at the concentration of 10 μg/mL (*p* < 0.001) and with AK at concentrations of 2.5, 5, 10 and 20 μg/mL (*p* < 0.001). Increases in the number of collagen granules determined with AL at concentrations of 2.5, 5, 10 and 20 μg/mL were not significant statistically (Figure 8).

In previous studies, wound-healing activities of *Achillea* species, such as *A. kellalensis* [4], *A. biebersteinii* [5], *A. millefolium* [6], have been demonstrated by experimental studies mainly based on animal models of excision and incision wounds. In the last few decades, *in vitro* bioassays for wound healing as a replacement for experiments using tissues or whole animals have been widely employed in ethnopharmacological research because of ethical reasons and their usefulness in bioactive-guided fractionation and determination of active compounds. They may be valuable also in providing evidence of the modes of action of plant materials [22]. In this fibroblast cell culture study, it was determined that findings related to TECA are in general agreement with previous cell culture studies [49,50,51]. TECA mainly heals wound by stimulation of fibroblast proliferation, migration and collagen synthesis. According to our results, among three *Achillea* species, AK strongly increased total fibroblast proliferation in NIH-3T3 cells (at concentrations between 2.5 and 20 μg/mL). Decreases with AK in the percentage of quiescent fusiform cells and increases in the percentage of active polygonal fibroblasts observed in this study showed that AK may also stimulate the migration of fibroblasts to close wounds. As an indicator of non-cytotoxic effect of AK, percentage of round fibroblasts and vacuole containing cells were found to be strongly decreased. AK also strongly stimulated the collagen production in the NIH-3T3 cells at low concentrations. These findings indicates possible effects of AK in the proliferation phase of wound healing which is characterized by fibroblast proliferation, migration and collagen synthesis. On the other hand, AC and AL were only increased collagen synthesis (at the concentration of 10 μg/mL) and stimulate fibroblast migration (at concentrations of 5 and 10 μg/mL), respectively. In this comparative fibroblast cell culture study, findings of all parameters indicated that AK has the most prominent wound healing potency whose mechanism of action is similar to TECA.

### 2.6. Cytotoxic Activity

**Figure 9:**
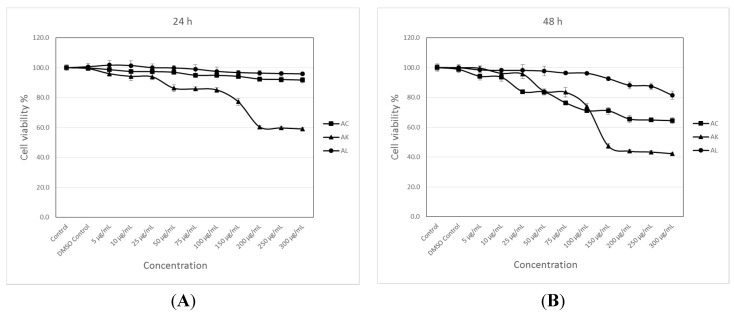
Cytotoxic effects of AC, AK and AL on MCF-7 cells estimated by the MTT reduction assay. Cells were treated with nothing (control, only medium), vehicle (DMSO, 0.1% in medium), and different concentrations of the extracts for 24 h (**A**); and 48 h (**B**). Data are mean ± SEM values (*n* = 8).

Cytotoxicity was assessed by the 3-(4,5-dimethylthiazol-2-yl)-2,5-diphenyltetrazolium bromide (MTT) assay, which is based on the reduction of MTT by the mitochondrial dehydrogenase of intact cells to a purple formazan product [52]. Percentage of cell growth with the different AC, AK and AL concentrations were calculated at the end of incubation for 24 and 48 h according to the dimethyl sulfoxide (DMSO) control group as it was used as a vehicle to dissolve extracts. For the solvent control group (DMSO), the percentages of cell growth values were between 99.4% and 100% (*p* > 0.05) in all assays. As shown in Figure 9, dose- and time-dependent statistically significant reductions in MCF-7 cell growth were observed in cells treated with AC, AK and AL. Statistically significant percentages of cell growth according to the DMSO control group with 200, 250 and 300 μg/mL AC concentrations after a 24-h incubation were found to be 92.4%, 92.2% and 91.8% (*p* < 0.01), respectively (Figure 9A). At 48 h, statistically significant decreases in MCF-7 cell growth with increasing AC concentrations from 25 μg/mL to 300 μg/mL were calculated to be 83.9%, 83.6%, 76.4%, 71.2%, 71.1%, 65.5%, 64.9% and 64.4% (*p* < 0.001), respectively (Figure 9B). At the end of the both 24- and 48-h incubation of MCF-7 cells with the increasing AK concentrations from 50 μg/mL–300 μg/mL gave us statistically significant reductions in cell growth which were calculated to be 86.3%, 85.8%, 85.1%, 77.2%, 60.4%, 59.7% and 59.0% (*p* < 0.001) at 24 h (Figure 9A), respectively, and 84.2%, 83.5%, 73.9%, 47.4%, 43.9%, 43.4% and 42.2% (*p* < 0.001) at 48 h, respectively (Figure 9B). Statistically significant growth values with AL at concentrations of 200, 250 and 300 μg/mL were found to be 88.1% (*p* < 0.05), 87.5% (*p* < 0.05) and 81.4% (*p* < 0.001), respectively, after a 48-h incubation (Figure 9B).

A literature survey revealed that anticancer potential of some *Achillea* species and their metabolites has been investigated *in vitro* using various tumor cell lines. Among them, 80% of ethanol extracts of *A. biebersteini* and *A. millefolium* exhibited cytotoxic effect against MCF-7 cells (IC_50_ = 47.468 μg/mL and 64.058 μg/mL, respectively) after 24 h of incubation [53]. In another study, cytotoxic activities of *N*-hexane, chloroform, aqueous-methanol and aqueous extracts of the aerial parts of the *A. millefolium* aggregate on MCF-7 cell lines were investigated by means of MTT assays and after 48 h incubation chloroform-soluble extract at 10 μg/mL exerted high tumor cell growth inhibitory activities on MCF-7 cells (53.95%) [54]. According to results of this study, among three *Achillea* species, methanol extract of AK exhibited the most abundant cytotoxic activity against MCF-7 cells after both 24- and 48-h treatment in a dose- and time-dependent manner, although it was not as effective as the above mentioned species.

In this comparative study, examination of total phenolic content and phenolic composition, *in vitro* studies on antioxidant, wound healing and cytotoxic potential of methanol extracts of AC, AK and AL were undertaken. Among three *Achillea* species, AK was found to be the richest species in terms of total phenolic content. A comparative LC-MS/MS study also demonstrated phenolic rich composition of AK. The most abundant flavonoid was hyperoside, followed by apigenin, hesperidin, rutin, kaempferol and luteolin in AK. Besides these flavonoids, a significant amount of chlorogenic acid was also evident. DPPH radical scavenging and total antioxidant capacity assays indicated that AK extract had the most powerful radical scavenging property and the highest total antioxidant capacity related with its phenolic content. AK also exhibited the most prominent wound healing potential without any toxicity at very small concentrations whose mechanism of action is similar to well-known wound healing agent TECA. In addition, the most significant cytotoxic activity against MCF-7 cells was observed with AK after both 24- and 48-h incubation periods.

Flavonoids and phenolic acids are known for their radical scavenging potential, and the position and degree of hydroxylation is fundamental to their antioxidant activity. In flavonoids, *O*-dihydroxy structure in the B ring, 2,3 double bond in conjugation with 4-oxofunction in the C ring and 3- and 5-OH moieties in a combination with 4-oxofunction in the A and C rings are required for maximum radical scavenging potential. Quercetin, which provides all the above mentioned determinants, and its glycosides, such as hyperoside and rutin, were reported to have significantly high antioxidant activity. In addition, apigenin, hesperidin, kaempferol, luteolin, and chlorogenic acid are also well-known phenolic compounds with their high antioxidant levels [11,55]. Furthermore, phenolic compounds also represented positive activity at different stages of the wound healing process by various mechanisms including antioxidant, antimicrobial, antiinflammatory, collagen synthesis stimulation, cell proliferative and angiogenic effect [51,56]. Major phenolic compounds of AK were previously reported to be active components of various herbal extracts such as *Hypericum perforatum*, *Helichrsyum graveolens* and *Martynia annua*, which exhibited strong wound healing activities [51,57,58]. Wound healing potential of many of these phenolics were also demonstrated in different experimental models [58,59,60], and the improving wound healing activity of phenolics were primarily explained by their antioxidant and free radical scavenger effects on oxidative parameters. Flavonoids and phenolic acids have also drawn increasing attention due to their marked effects in cancer [11,61]. Previous studies have demonstrated that chlorogenic acid, hyperoside, apigenin, hesperidin, rutin, kaempferol and luteolin, which were found in AK in significant amounts, are potent cytotoxic agents against various cancer cell lines [11,54,61,62,63].

To the best of our knowledge, this is the first report describing the phenolic composition, antioxidant, wound healing and cytotoxic potential of methanol extracts of AC, AK and AL. Thus, significant activities of AK may be attributed to the phenolic constituents present in the aerial parts, which may be either due to their individual or combined effect. In addition, synergistic interaction with the rest of the ingredients might also promote activities.

## 3. Experimental Section

### 3.1. Plant Materials

The plants were collected during the flowering stage from different provinces of inner Anatolia. The collected samples were identified and the voucher specimens were deposited in the Herbarium of Hacettepe University, Faculty of Pharmacy (HUEF). Collection sites and herbarium codes of the plants are given as follows; *A. coarctata* Poir. (AC): B5 Nevşehir: Nevşehir to Aksaray, 2 km to Camiören village, 38°33ʹ35ʹʹ K, 34°23ʹ36ʹʹ D, 1296 m, roadside, 20.06.2009 (HUEF 09873); *A*. *kotschyi* Boiss. subsp. *kotschyi* (AK): B6 Yozgat: Akdağmadeni, above Kızılcaova village, Nalbant hill, 39°32ʹ37ʹʹ K, 36°00ʹ47ʹʹ D, 2080 m, Alpine meadows, 17.07.2009 (HUEF 09867); *A. lycaonica* Boiss. & Heldr. (AL): B6 Sivas: Ulaş, Ziyarettepe, 39°34ʹ00ʹʹ K, 37°01ʹ10ʹʹ D, 1450 m, gypseous areas, 18.07.2009 (HUEF 09864).

### 3.2. Extraction Procedure

The aerial parts of the plants were dried in shade at room temperature and ground in a mechanic grinder to fine powder and weighed accurately (Schimadzu, Kyotoo, Japan). The powdered plant material were extracted with methanol (100%) (Sigma-Aldrich, St. Louis, MO, USA) on a hot plate at 40 °C mixing with a magnetic stirrer. Methanol extract of each plant material was filtered through regular filter paper and evaporated under reduced pressure (HeidolphLaborota 4003, Schwabach, Germany) until dryness to give crude extract.

### 3.3. Identification and Qualification of Phenolic Compounds by LC-MS/MS

In order to identify and quantify phenolic compounds, a LC-MS/MS method, which was prevously reported by our research group, was used [31,32]. Applicability of the analytical method and the qualitative and quantitative determination of the standard compounds have already been verified. Rectilinear regression quotations and the linearity ranges of the studied standard compounds were given in Table 2. Correlation coefficients were found to be higher than 0.99. The limit of detection (LOD) and the limit of quantitation (LOQ) of the reported analytical method were shown in Table 2. For the studied compounds, LOD ranged from 0.05–25.8 µg/L and LOQ ranged from 0.17–85.9 µg/L. Moreover, the recoveries of the phenolic compounds ranged from 96.9% to 106.2%.

### 3.4. Determination of Total Phenolic Content

Folin-Ciocalteu (Sigma-Aldrich) colorimetric method [33] using gallic acid (Sigma-Aldrich) as a standard phenolic compound gives a crude estimation of the total phenolic compounds by measuring the amount of the substance being tested needed to inhibit the oxidation of the reagent. Briefly, 0.5 mL of the extracts (0.5 mg/mL), 2.5 mL Folin-Ciocalteu reagent solution (10% *v*/*v* in water) and 7.5 mL of saturated sodium carbonate (Merck, Darmstadt, Germany) (20% *w*/*v*, water) were added into a test tube. The absorbance of the resulting blue-coloured solution was measured at 750 nm after incubation at 30 °C for 2 h with intermittent shaking. Total phenolic content was expressed as gallic acid equivalents (GAE) in milligram per grams of dry material.

### 3.5. Antioxidant Activity

#### 3.5.1. DPPH Radical Scavenging Assay

The free radical scavenging activity of the fractions was measured *in vitro* by 2,2ʹ-diphenyl-1-picrylhydrazyl (DPPH) assay according to the method described earlier [39]. The stock solution was prepared by dissolving 24 mg DPPH (Sigma-Aldrich) with 100 mL methanol and stored at 20 °C until required. The working solution was obtained by diluting DPPH solution with methanol to attain an absorbance of about 0.98 ± 0.02 at 517 nm using the spectrophotometer. A 3 mL aliquot of this solution was mixed with 100 μL of the sample at various concentrations (1–150 μg/mL). The reaction mixture was shaken well and incubated in the dark for 30 min at room temperature. Then, the absorbance was taken at 517 nm. The scavenging activity was estimated based on the percentage of DPPH radical scavenged as the following equation:

DPPH radical scavenging activity (%) = (A_0_ − A_1_)/A_0_ × 100 (1)

A_0_: Absorbance of the control at 30 min (517 nm), A_1_: Absorbance of the sample at 30 min (517 nm).

BHT (Sigma-Aldrich) was used as a positive control. Tests were carried out in triplicate. Afterwards, a curve of % DPPH scavenging capacity *versus* concentration was plotted and EC_50_ values were calculated. EC_50_ denotes the concentration of sample required to scavenge 50% of DPPH free radicals.

#### 3.5.2. β-Carotene-Linoleic Acid Assay

A stock solution of β-carotene linoleic acid mixture was prepared as follows: 3 mg of β-carotene (Sigma-Aldrich) was dissolved in chloroform and it was added to 40 mg of linoleic acid (Sigma-Aldrich) and 400 mg of Tween 80 (Merck, Kenilworth, NJ, USA). Chloroform (Merck) was gently removed by using a rotary evaporator. Then, 100 mL of distilled water, saturated with oxygen, was added slowly to the residue and the solution was vigorously agitated to form a stable emulsion. To an aliquot of 3 mL of this emulsion, 0.2 mL of sample solutions (0.6 mg/mL concentration) was added and each sample solution was transferred to a 96-well microplate. Absorbance was taken at 490 nm after incubation for every 15 min until 180 min at 45 °C using a microplate reader. BHT was used as a reference synthetic antioxidant. An equal amount of methanol was used as control [40]. All determinations were performed in triplicate and results were averaged. The percentage inhibition was calculated using the following equation:

Inhibition (%) = [[1 − (As_0_ − As_180_)/(Ac_0_ − Ac_180_)] × 100 (2)

As_0_: Absorbance of the sample at 0 min (490 nm), As_180_: Absorbance of the sample at 180 min (490 nm), Ac_0_: Absorbance of the control at 0 min (490 nm) , Ac_180_: Absorbance of the control 180 min (490 nm).

#### 3.5.3. Total Antioxidant Capacity (TAC) Assay

The TAC assay is based on the reduction of copper (II) to copper (I) by a wide variety of antioxidants [41]. The assay was carried out using commercial TAC assay kit (OxiSelect™ Total Antioxidant Capacity (TAC) Assay Kit, Cell Biolabs, Inc., San Diego, CA, USA). Upon reduction, the copper (I) ion further reacts with a coupling chromogenic reagent that produces a color with a maximum absorbance at 490 nm. The net absorbance values of antioxidants are compared with a known uric acid standard curve. Absorbance values are proportional to the sample’s total reductive capacity. Results are expressed as μM copper reducing equivalents or mM uric acid equivalents. A fresh uric acid standard was prepared by weighing out the uric acid powder for a 10 mg/mL solution in 1 N NaOH. This 10 mg/mL is equivalent to a concentration of 60 mM. The 60 mM uric acid solution was used to prepare a 2 mM solution of uric acid (e.g., add 100 μL of the 60 mM uric acid standard to 2.900 μL of deionized water). Each sample was prepared using the stock solution of 10 mg/mL concentration. An initial reading was taken at 490 nm. Then, 50 μL of the 1× copper ion reagent was added and incubated for 5 min on an orbital shaker. Then, 50 μL of the stop solution was added to terminate the reaction and the plate was read again at 490 nm. All determinations were performed in triplicate and results were averaged.

### 3.6. Determination of Wound Healing Potency

#### 3.6.1. Cell Culture and Treatment

Wound healing activity of extracts was evaluated by comparing with titrated extract of *Centella asiatica* (TECA, which was a gift from Bayer Chemical Industry, Istanbul, Turkey) on NIH-3T3 mouse fibroblast cells which were proposed as a method for testing wound-healing activity *in vitro*. The NIH-3T3 mouse fibroblast cells (ATCC number CRL-1658) were grown in Dulbecco’s Modified Eagle Medium (DMEM) (Sigma-Aldrich) medium supplemented with 2 mm l-glutamine (Biochrom, Berlin, Germany) and 10% fetal bovine serum (Biological Industries, Beit-Haemek, Israel), 1% penicillin/streptomycin (Biochrom) at a temperature of 37 °C in a humidified incubator with a 5% CO2 atmosphere. TECA and AC, AK and AL extracts were dissolved in DMSO (Sigma-Aldrich) at a stock solution and the stock solution was diluted to the required concentrations in cell medium. The same dose of DMSO at 2 μL/mL was used for the DMSO control. Results of experimental groups were compared with DMSO control. The cell monolayers were grown on cover slips. A total of 70%–80% confluent cells were treated with TECA and AC, AK and AL extracts (2.5, 5, 10, 20 and 40 μg/mL) for 24 h in growth medium [51].

#### 3.6.2. Staining and Morphometric Analysis of Cultured NIH-3T3 Fibroblasts

HT15-1KT Trichrome Stain (Masson) Kit (Sigma-Aldrich) were used for the staining of cultured NIH-3T3 fibroblasts. Morphometric analysis was carried out according to previously described procedure [49,50,51]. After staining, the controls and extract treated fibroblasts were examined under a light microscope (Olympus, Tokyo, Japan) using a Neubauer slide (×400 magnification). Total cell numbers and cell numbers under mitosis are used as parameters of proliferation of fibroblasts. Fusiform, polygonal, round and vacuole containing fibroblasts, whose morphological shapes reflect functional states of cells, were expressed as a percentage of the total cell number (cells seeded initially at a density of 2 × 10^5^). For each experimental group, at least three visible areas were counted and results were reported as the mean of five individual experiments. The number of collagen granules in a cell was also counted in at least 10 cells of five individual experiments.

### 3.7. Determination of Cytotoxic Activity

#### 3.7.1. Cell Culture and Treatment

MCF-7 human breast adenocarcinoma cells were grown in a RPMI-1640 (Biochrom) medium supplement including 10% fetal bovine serum (Biological Industries), 1% penicillin-streptomycin (Biochrom), 1% l-glutamine (Biochrom), 1% gentamicin (Biochrom) at a temperature of 37 °C in a humidified incubator with a 5% CO_2_ atmosphere. *Achillea* methanol extracts dissolved in dimethyl sulfoxide (DMSO) (Sigma-Aldrich) at a stock solution, and the stock solution was diluted to the required concentrations in cell medium. A total of 70%–80% confluent cells (after 24 h) were treated with extracts (concentrations of 25, 50, 100, 200, and 300 μg/mL) for 24 and 48 h in growth medium [64].

#### 3.7.2. Cytotoxicity/Cell Proliferation Assay

MCF-7 cells were seeded at a density of 3 × 10^4^ cells/mL onto a 96-well microplate. After overnight incubation at 37 °C in 5% CO_2_ atmosphere, they were treated with AC, AK and AL (concentrations of 5, 10, 25, 50, 75, 100, 150, 200, 250 and 300 µg/mL) for 24 and 48 h. After incubations, MTT solution (Sigma-Aldrich) (0.5 mg/mL) was added to each well and incubated at 37 °C in 5% CO_2_ atmosphere (Hera Cell 240 Thermo Scientific, Waltham, MA, USA) for 3 h. At the end of the incubations, the purple MTT-formazan crystals were dissolved by adding 100 μL of dimethyl sulfoxide to each well. The absorbance of the samples was measured with microplate reader (BioTek Power Wave XS, Winooski, VT, USA) (wavelength of 540 nm). In the experiment, each group was tested in eight wells. The data are mean values from three different experiments. Cytotoxic effect of AC, AK and AL against MCF-7 cancer cells was expressed as relative cell viability, using the following formula:

Percentage of cell viability = (optical density of drug-treated sample/optical density of untreated sample) × 100 [64].

### 3.8. Statistical Analysis

The results were reported as means ± standard deviations and standard errors of mean (SEM). Statistical differences between the experimental groups were determined by one-way analysis of variance (ANOVA) followed by *post-hoc* Tukey’s tests using IBM SPSS Statistics for Windows, Version 22.0 (IBM Corp., Armonk, NY, USA, 2013). Differences were considered statistically significant if *p* < 0.05.

## 4. Conclusions

The results of the present study provide important data regarding phenolic composition, antioxidant, wound healing and cytotoxic potential of methanol extracts from *A. coarctata*, *A. kotschyi* subsp. *kotschyi* and *A. lycaonica*. Among three species, phytochemical investigations reveal *A. kotschyi* subsp. *kotschyi* as a valuable source of flavonoids such as hyperoside, apigenin, hesperidin, rutin, kaempferol and luteolin, and chlorogenic acid. In terms of total phenolics, *A. kotschyi* subsp. *kotschyi* extract contains distinctively higher amounts. The antioxidant potential of three species was tested through several assays, which indicated *A. kotschyi* subsp. *kotschyi* exhibited very strong DPPH free radical scavenging activity and high total antioxidant capacity. Furthermore, *A. kotschyi* subsp. *kotschyi* exhibited a very prominent wound healing potency at very small concentrations whose mechanism of action is similar to the well-known wound healing agent TECA, and also exhibited moderate cytotoxic activity against MCF-7 cells. Summarizing the results of the present research, we can conclude that aerial parts of *A. kotschyi* subsp. *kotschyi* are a valuable source of flavonoids and chlorogenic acid with important antioxidant, wound healing and cytotoxic activities. Therefore, *A. kotschyi* subsp. *kotschyi* could be used in the development of new pharmaceuticals, cosmetics as well as food products and additives. However, further studies, particularly *in vivo* tests, are needed to understand its activities in biological systems.
